# Dropout Rate of Participants with Cancer in Randomized Clinical Trials That Use Virtual Reality to Manage Pain—A Systematic Review with Meta-Analysis and Meta-Regression

**DOI:** 10.3390/healthcare13141708

**Published:** 2025-07-16

**Authors:** Cristina García-Muñoz, María-Dolores Cortés-Vega, Patricia Martínez-Miranda

**Affiliations:** 1 Departamento de Ciencias de la Salud y Biomédicas, Faculta de Ciencias de la Salud, Universidad Loyola Andalucía, 41704 Seville, Spain; cgmunoz@uloyola.es (C.G.-M.); pmartinez@uloyola.es (P.M.-M.); 2 CTS 1110: Understanding Movement and Self in Health from Science (UMSS) Research Group, 41009 Andalusia, Spain; 3 Department of Physiotherapy, University of Seville, 41009 Seville, Spain

**Keywords:** cancer, pain management, systematic review, virtual reality, meta-analysis, dropout

## Abstract

**Background/Objectives**: Virtual reality has emerged as a promising intervention for pain management in individuals with cancer. Although its clinical effects have been explored, little is known about participant adherence and dropout behavior. This systematic review and meta-analysis aimed to estimate the pooled dropout rate in randomized controlled trials using virtual reality to treat cancer pain; assess whether dropout differs between groups; and explore potential predictors of attrition. **Methods**: We conducted a systematic search of PubMed, Web of Science, Scopus, and CINAHL up to April 2025. Eligible studies were randomized trials involving cancer patients or survivors that compared VR interventions for pain management with any non-VR control. Proportion meta-analyses and odds ratio meta-analyses were performed. Heterogeneity was assessed using the I^2^ statistic, and meta-regression was conducted to explore potential predictors of dropout. The JBI appraisal tool was used to assess the methodological quality and GRADE system to determine the certainty of evidence. **Results**: Six randomized controlled trials were included (n = 569). The pooled dropout rate was 16% (95% CI: 8.2–28.7%). Dropout was slightly lower in VR groups (12.7%) than in controls (21.4%), but the difference was not statistically significant (OR = 0.94; 95% CI: 0.51–1.72; I^2^ = 9%; GRADE: very low). No significant predictors of dropout were identified. **Conclusions**: VR interventions appear to have acceptable retention rates in oncology settings. The pooled dropout estimate may serve as a reference for sample size calculations. Future trials should improve reporting practices and investigate how VR modality and patient characteristics influence adherence.

## 1. Introduction

Cancer is an ongoing global health challenge that affects individuals of all ages, including adults and children. Millions of new cancer cases are diagnosed annually worldwide, highlighting the pervasive nature of this disease. Pain is a significant and distressing symptom associated not only with the malignancy itself but also with adverse effects of treatments such as chemotherapy. Additionally, pain is common among cancer survivors, often persisting long after active treatment completion [1,2,3,4]. This persistent pain may result from surgical interventions, radiation-induced tissue damage, chemotherapy-induced peripheral neuropathy, and chronic inflammation. A global study reported that over 35% of cancer survivors experience moderate-to-severe pain years after diagnosis and treatment, underscoring the long-term burden of pain even during remission phases [5]. Epidemiological data indicate that approximately 44.5% of cancer patients experience pain, with moderate-to-severe pain reported by 30.6%, profoundly impacting physical functioning, emotional well-being, and quality of life [4].

Although children and adults differ in their physiological development and cognitive processing, both groups can experience cancer-related pain with significant emotional and functional impact. While pediatric patients may show greater sensory sensitivity and shorter attention spans, virtual reality has been shown to be well-tolerated and engaging across age groups. Given the limited number of randomized controlled trials available, we included both populations to provide a more comprehensive overview, while acknowledging this as a source of clinical heterogeneity.

Pharmacological interventions have traditionally been the cornerstone of cancer pain management; however, non-pharmacological strategies are gaining increasing recognition for their potential benefits. Among these, virtual reality (VR) has emerged as a promising therapeutic modality. VR technology immerses users in computer-generated environments, engaging multiple senses and diverting attention from painful stimuli. This immersive experience can be delivered through various modalities, including head-mounted displays and projection-based systems. The application of VR in pain management leverages mechanisms such as distraction, neuroplasticity, and modulation of pain perception pathways, offering potential benefits for cancer patients [6,7].

Recent literature has explored VR’s efficacy in alleviating cancer-related pain, demonstrating significant pain reductions in hospitalized cancer patients [8,9]. Despite these promising results, research on patient adherence to VR interventions compared to other pain management strategies remains scarce. Understanding patterns and reasons for participant attrition in VR-based studies is critical, as dropout threatens both internal and external validity in research and clinical settings. High attrition rates can compromise study validity and impede effective implementation of VR interventions in practice. Analyzing dropout rates and factors contributing to participant loss is essential for developing strategies to enhance adherence and optimize treatment outcomes [10].

Given the expanding interest in VR as a non-pharmacological intervention for cancer pain management, this systematic review and meta-analysis aims to meta-analyze pooled dropout rates in randomized controlled trials involving VR interventions for cancer pain, compare dropout likelihood between VR and comparator groups, and examine potential predictors of attrition.

## 2. Materials and Methods

### 2.1. Data Sources and Search Strategy

This systematic review followed the Preferred Reporting Items for Systematic Reviews and meta-analyses statements of 2020 (PRISMA 2020) [11]. The review protocol was registered in the OSF registry with https://doi.org/10.17605/OSF.IO/XQKE2.

Two independent reviewers (PMM and CGM) conducted independent searches of PubMed, Web of Science, CINAHL, and Scopus from their inception to April 2025. The following search terms were used and combined using AND/OR Boolean operators in the different databases (PubMed, Web of Science, CINAHL and Scopus): cancer, neoplasm, virtual reality, pain, and pain management. Supplementary File S1 shows an extended search strategy.

### 2.2. Research Question and Study Selection

The eligibility criteria were established using the PICOS model (Population, Intervention, Comparator, Outcomes, and Study design). The inclusion criteria were as follows:

- P: patients diagnosed with cancer or survivors of cancer (adults and pediatric patients).
- I: virtual reality-based interventions related to pain management.
- C: all types of comparators, except those based on virtual reality
- O: participant dropout.
- S: randomized clinical trials that report the dropout rate or allow its indirect calculation.
- The exclusion criteria were as follows:
- Studies in which the comparator is also a virtual reality group because comparisons could not be conducted.
- Studies with the same sample size as other publications.

### 2.3. Data Extraction

The reviewers who conducted the literature search were also responsible for screening titles and abstracts to identify potentially relevant records. Once potential studies had been selected, duplicate entries were eliminated using Mendeley Desktop (version 1.19.8). Full-text articles were then reviewed to assess whether they fulfilled the established eligibility criteria. In cases where disagreements arose, a third reviewer (MDCV) was consulted to reach consensus.

To organize the data for the qualitative synthesis, a structured extraction table was used, while for the quantitative synthesis, we compiled the data using an Excel spreadsheet (version 2207). The qualitative synthesis included information such as author names, sample sizes, type of cancer, age, interventions, overall retention rates, dropout rates, reasons for withdrawal, and adverse events.

For the quantitative analysis, the following variables were extracted: study identifier, participant age, gender distribution, type of virtual reality intervention, immersion level, number and frequency of sessions, session duration, intervention length (in weeks), dropouts in both experimental and control groups, total sample size, and overall dropout. All included articles provided sufficient data to compute dropout rates; therefore, it was not necessary to contact the corresponding authors.

### 2.4. Data Analysis

We defined a dropout as any participant who failed to complete the intervention or the follow-up period after being randomized. In studies involving more than two intervention arms, comparisons between groups were carried out in a pairwise manner.

For the statistical analyses, we used RStudio (version 4.1.0) along with the metafor (v3.0-2), meta (v5.1-1), and dmetar (v0.0.9000) packages. All meta-analyses were performed using a random-effects model to account for heterogeneity among the included randomized controlled trials. The results were illustrated using forest plots.

To estimate dropout rates, a proportion meta-analysis was performed, calculating both the overall pooled dropout rate and the rate per intervention group. Proportions were logit-transformed for analysis. In studies reporting zero dropouts, a continuity correction of 0.5 was applied. We also conducted a meta-analysis using odds ratios (ORs) to compare dropout likelihoods between groups. An OR < 1 indicated that participants receiving virtual reality interventions had lower dropout rates. We reported 95% confidence intervals (CIs) and applied inverse variance methods to adjust for sparse data. Tau^2^ was estimated using the restricted maximum likelihood (REML) approach to capture between-study variance. Heterogeneity was quantified using the I^2^ statistic, considering values above 50% as indicative of substantial variability.

To assess potential outliers, we conducted a sensitivity analysis, which included generating Baujat plots, L’Abbé plots, leave-one-out diagnostics, and influence plots. Studies flagged as influential, or outliers, were excluded from the final meta-analysis to avoid biased estimates. Publication bias was examined using a contour-enhanced funnel plot, along with the trim-and-fill method and Egger’s tests (presence of publication bias when *p* < 0.05).

To explore potential factors contributing to dropout, we ran a univariate mixed-effects meta-regression. Predictors included the type of virtual reality intervention, session characteristics (number, frequency, and total weeks), participant sex and age, sample size, VR immersion level, and chemotherapy. As in the subgroup analyses, meta-regression was only performed when at least three studies contributed and there were non-redundant data for a given predictor.

## 3. Results

### 3.1. Study Selection

**Figure 1:**
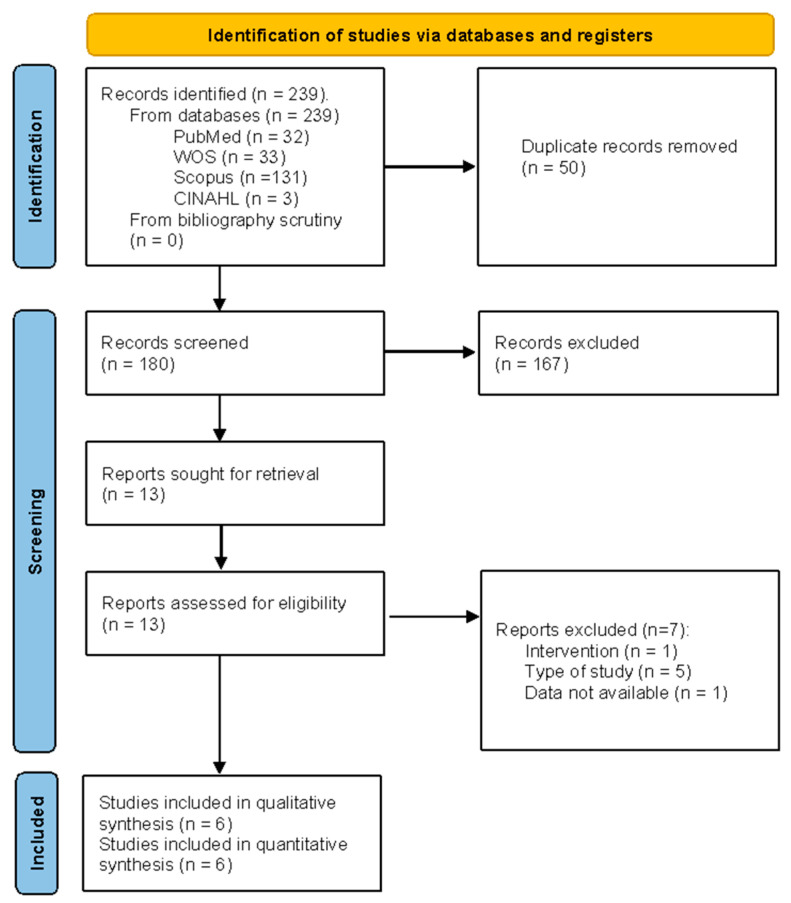
Flow diagram.

The selection process was performed in accordance with the PRISMA recommendations. Finally, six studies [12,13,14,15,16,17] were included in the qualitative and in the quantitative synthesis. Figure 1 and Supplementary File S2 contain more detailed descriptions of the selection process.

### 3.2. Methodological Quality Assessment: JBI Critical Appraisal Tool

Table 1 shows the results for the JBI critical appraisal tool. Overall, the methodological quality of the studies included was good. Of the 78 items assessed, 64 were given a favorable score (“yes”), 12 received an unfavorable score (“no”) and 2 were assigned an uncertain score. All of the included studies used a randomization method, with identical treatment between groups apart from the intervention of interest, and they all employed blinding of the assessors, good data presentation, and follow-up, and all followed a pre-established protocol. In addition, all of the included studies aside from that of Erdős et al., 2023 [14] were controlled. All studies had similar treatment groups at the baseline, except two of them, for which this item was classed as uncertain [13,16]. The items with the highest risk of bias were those related to participant and therapist blinding, with the study of Abdelmoniem et al., 2024 [12] being the only one that employed blinding of participants. The percentage of discrepancy was 0%.

### 3.3. Description of the Selected Studies

A total of six studies [12,13,14,15,16,17] were included in the qualitative synthesis, with a sample of 572 people diagnosed with cancer that reported suffering from pain. Most of the participants (4/6) were adult breast cancer patients, while one of them [14] was a pediatric cancer patient, and another [16] had been diagnosed with adult advanced cancer. All of the samples, except that in the study by Erdős et al., 2023 [14], were predominantly composed of women (female n = 522; male n = 50). The medium age in the studies oscillated between 47 and 55 years, except for the study that focused on a pediatric population [14] (medium age: 15 ± 2.44). It was noted that 66.67% of the interventions were based on the application of virtual reality alone, while 33.33% combined virtual reality with other therapies (usually standard physical therapy or exercise). The duration of the intervention ranged from 4 days to 12 weeks and the medium duration was 8.5 weeks. Regarding the comparison, 50% of the studies utilized other forms of therapy, such as standard exercise or physical therapy; 33.33% used other types of technology such as mobile games or tablets; and 16.67% applied the usual care. Most of the studies achieved greater than 75% retention, with less than 25% of participants dropping out, except for the study by Abdelmoniem et al., 2024 [12], for which the following percentages were reported: 74.05% and 25.93%, respectively. The overall number of dropouts was 118. The majority of the studies (66.67%) provided reasons for dropouts and adverse events. None of them reported any adverse events. More details are shown in Table 2 and Supplementary File S3.

### 3.4. Sensitivity Analysis and Publication Bias

None of the assessed randomized controlled trials were identified as outliers; because of this, no study was disregarded. There was no consistency between the exploratory analyses, so we decided not to discard any of the included studies. All sensitivity analyses can be found in Appendix A.

**Figure 2:**
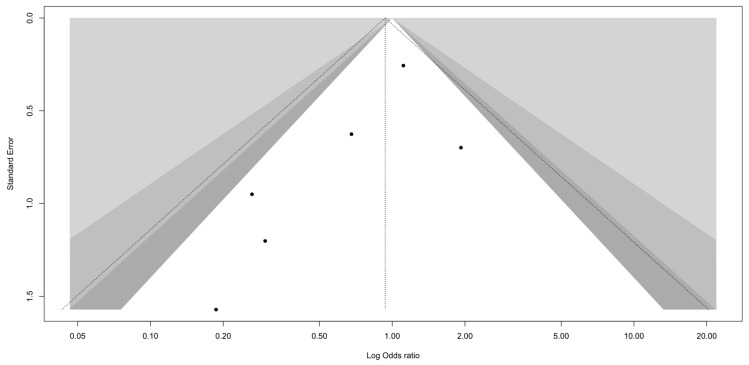
Funnel plot.

A visual inspection of the funnel plot did not reveal substantial asymmetry (Figure 2). Egger’s test of small-study effects showed no evidence of publication bias (*p* = 0.118). Given the small number of included studies (n = 6), the test may have limited power, and the results should be interpreted with caution.

### 3.5. Proportion Meta-Analysis

**Figure 3:**
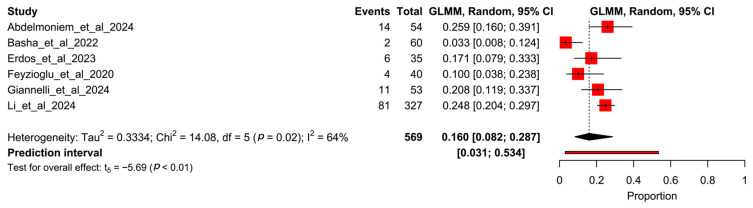
Forest plot of proportion meta-analysis from all arms of studies. Abdelmoniem et al., 2024 [12], Basha et al., 2022 [13], Erdős et al., 2023 [14], Feyzioğlu et al., 2020 [15], Giannelli et al., 2024 [16], Li et al., 2024 [17].

A total of six randomized controlled trials were included in these meta-analyses. The proportion meta-analysis for all participants within the studies reported a pooled rate of 16% (95% CI 8.2–28.7%, I^2^ = 64%), comprising a total of 118 dropouts out of 569 participants (Figure 3).

**Figure 4:**
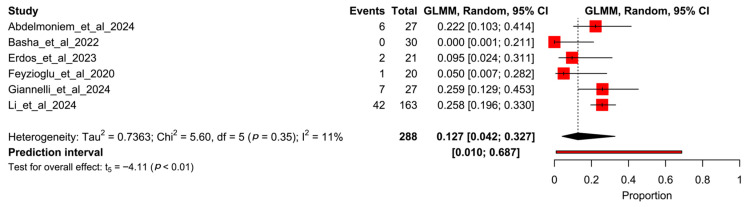
Forest plot of proportion meta-analysis from experimental arms of studies. Abdelmoniem et al., 2024 [12], Basha et al., 2022 [13], Erdős et al., 2023 [14], Feyzioğlu et al., 2020 [15], Giannelli et al., 2024 [16], Li et al., 2024 [17].

Figure 4 shows the forest plot for the pooled dropout rate in those participants who received the VR-based intervention. The pooled rate is 12.7% (95% CI 4.2–32.7%, I^2^ = 11%), representing 58 withdrawals out of 288 participants.

**Figure 5:**
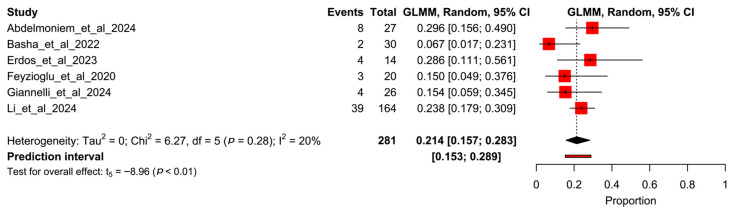
Forest plot of proportion meta-analysis from control arms of studies. Abdelmoniem et al., 2024 [12], Basha et al., 2022 [13], Erdős et al., 2023 [14], Feyzioğlu et al., 2020 [15], Giannelli et al., 2024 [16], Li et al., 2024 [17].

Figure 5 shows the forest plot for the control participants, revealing a total of 60 dropouts out of 281 participants, with a pooled rate of 21.4% (95% CI 15.7–28.9%, I^2^ = 64%).

### 3.6. Odds Ratio Meta-Analysis

**Figure 6:**
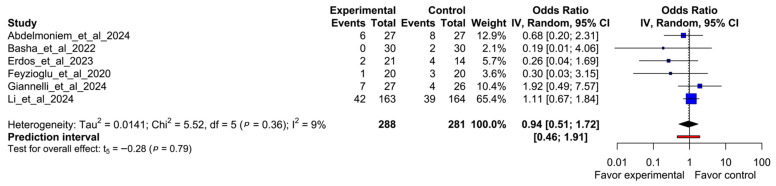
Forest plot of odds ratio comparing participant dropout between virtual reality-based intervention and comparators. Abdelmoniem et al., 2024 [12], Basha et al., 2022 [13], Erdős et al., 2023 [14], Feyzioğlu et al., 2020 [15], Giannelli et al., 2024 [16], Li et al., 2024 [17].

The overall odds ratio meta-analysis showed no statistically significant difference for the likelihood of the event occurring between experimental and comparator interventions (OR 0.94; 95% CI, 0.51–1.72; I^2^ = 9%; *p* = 0.36) (Figure 6).

### 3.7. Subgroup Meta-Analysis and Meta-Regressions

Supplementary File S3 shows the forest plots from the different subgroup analyses. None of the subgroups showed a significant difference in the likelihood of dropouts.

Table 3 displays the meta-regression results, reporting that none of the analyzed predictors modified the pooled results.

### 3.8. Evidence Synthesis

Table 4 shows the evidence synthesis using the GRADE system. Due to the high heterogeneity and the possibility of bias, the certainty of evidence was rated as “very low”.

## 4. Discussion

This systematic review and meta-analysis explored dropout rates in randomized controlled trials using virtual reality for pain management in patients with cancer. We included six randomized controlled trials after the search, screening, and final selection. The pooled dropout rate across all groups was 16%, with slightly lower rates in VR-based interventions (12.7%) compared to controls (21.4%). However, the odds ratio meta-analysis did not reveal a statistically significant difference between groups, and the subgroup and meta-regression analyses failed to identify predictors of dropout. Overall, the certainty of evidence was rated as very low according to the GRADE system, which limits the strength of the conclusions. The high heterogeneity found between studies in terms of population, objective, and interventions should be considered when interpreting the results. In the next few paragraphs, we will discuss some of these issues.

Among the studies included, the main reasons for dropout were physical complications related to cancer (e.g., side effects from chemotherapy, new metastatic lesions), loss of interest, and personal decisions such as declining to participate. However, only four of the six studies provided specific reasons for attrition. This gap significantly limits our ability to analyze dropout patterns and develop informed retention strategies. In contrast, previous research in oncology trials has systematically identified fatigue, worsening clinical condition, adverse effects of treatment, and logistical barriers as major reasons for attrition [18,19,20,21]. The lack of detailed dropout data in one-third of the included studies in our review underscores the need for better adherence to reporting standards. Future studies should explicitly describe the reasons for participant dropout and the exact timing of the dropout (e.g., during intervention or the follow-up phase), enabling improved understanding and prevention of attrition.

Additionally, adverse events specifically related to VR-based interventions were insufficiently described across most studies. While cancer-related symptoms such as fatigue or pain are well-known factors that contribute to dropout, adverse effects directly caused by VR, such as cybersickness, should also be monitored and reported. Symptoms like nausea, dizziness, and visual discomfort can also occur in immersive VR systems, potentially affecting adherence and outcomes [22,23]. Only one of our included studies [14] considered this possibility, opting for a non-moving immersive VR environment to reduce sensory conflict between visual and vestibular cues. This proactive adaptation illustrates how technical design choices can mitigate risks and enhance participant tolerance. Future trials should integrate structured monitoring protocols for intervention-specific side effects and report them transparently to ensure patient safety and intervention feasibility.

Despite the relatively low heterogeneity observed in the odds ratio meta-analysis (I^2^ = 9%), significant clinical and methodological heterogeneity was evident across the included studies. Differences in participant demographics (e.g., adults vs. children, cancer type, disease stage, surgical status), intervention content (immersive vs. non-immersive VR), and implementation parameters (session frequency, duration, total intervention length) complicate direct comparisons and suggest the need for caution when interpreting pooled outcomes. Furthermore, there was heterogeneity in the objectives of the included trials, with some focusing on physical rehabilitation, while others focused on relaxation or symptom management, highlighting the diverse approaches to VR-based interventions in oncology. These are some of the reasons why the certainty of our findings was rated as “very low” using the GRADE system. This rating was primarily driven by high risk of bias, mainly due to lack of blinding of participants and therapists, and by substantial clinical and methodological heterogeneity across studies, including differences in cancer type, VR modality, and intervention objectives.

Given the variability in technological approaches across studies, there is a clear need to further investigate the distinct modalities of virtual reality used for pain management in patients with cancer. VR interventions range from non-immersive formats (e.g., screen-based experiences) to fully immersive environments employing head-mounted displays, each offering specific advantages and potential limitations. Immersive VR, for example, can produce stronger analgesic effects by inducing a greater sense of presence and attentional distraction from pain stimuli, particularly in acute or procedural pain settings [24,25]. In contrast, non-immersive or semi-immersive VR may be more suitable for individuals with physical limitations, sensory sensitivity, or vulnerability to cybersickness [26]. Despite these potential differences, few oncology trials have systematically compared these modalities. Future research should address this gap by exploring not only the comparative effectiveness of VR types for pain relief, but also how specific modalities may influence dropout rates and participant adherence. Understanding the tolerability and acceptability of each VR format across patient subgroups is essential to optimize retention strategies and intervention feasibility. In addition, the heterogeneity between control comparators complicates interpretation of treatment effects and dropout behavior. Harmonized approaches to control design would support more consistent evaluation and enable higher-quality evidence synthesis in future meta-analyses [27,28].

In our review, population heterogeneity also poses a significant barrier to drawing solid conclusions. The included studies involved both pediatric and adult participants, with varying cancer types, stages, and treatment contexts. These differences inherently influence symptom burden, psychological responses, and engagement with digital interventions. For instance, children and adolescents may respond differently to VR experiences compared to older adults due to variations in attention span, cognitive flexibility, and familiarity with immersive technologies [27,29]. Similarly, the nature of cancer pain, whether it is post-surgical, treatment-related, or chronic, varies substantially across diagnoses and disease stages, affecting both intervention suitability and dropout risk. This clinical and demographic diversity introduces substantial variability that limits the generalizability of our findings. To strengthen future research, trials should either be stratified by population subgroup or focus on more narrowly defined cohorts, thereby improving the clarity and applicability of outcomes related to retention and intervention feasibility.

Beyond the type of VR used, specific design features such as level of interactivity, ease of use, and potential for cybersickness may significantly impact adherence. Poor usability or sensory discomfort can lead to disengagement, especially in vulnerable patients. These factors should be carefully considered when developing VR interventions and reported systematically in future studies. Among the included studies, immersive VR interventions [14,16,17] tended to report reductions in pain intensity during chemotherapy or advanced cancer care, although these effects were not always directly quantified or comparable due to differences in study design. Non-immersive approaches [12,13,15] often focused on physical rehabilitation outcomes, where pain relief was a secondary benefit. However, no included trial systematically compared VR modalities head to head for pain reduction. These observations reinforce the need for future trials to explore how immersion level and content type influence not only retention, but also therapeutic efficacy.

### 4.1. Research and Clinical Implications

This heterogeneity is not exclusive to cancer populations. Similar variability has been reported in VR trials targeting pain in non-cancer contexts, where the diversity in intervention design, target populations, and outcome measures also limits the synthesis of findings and clinical translation [30,31]. A recent review by Garland et al. [30] on immersive VR for acute and chronic pain across different conditions revealed inconsistencies in dosage, content, and delivery modalities, which hinder generalization. Moreover, the lack of consensus on core outcomes and standard protocols has been identified as a persistent barrier to advancing VR pain research [31]. This pattern is echoed in oncology settings, where even within cancer-specific populations, studies show wide divergence in goals, methodologies, and metrics [32].

These issues underscore the urgent need for greater standardization and harmonization in the design and reporting of VR-based trials. As interest in this field grows, a more structured, hypothesis-driven approach, ideally following established guidelines such as CONSORT [33], is essential to improve comparability across studies and advance the evidence base for clinical implementation. This includes detailing the number of participants lost, the specific reasons for dropout, and the timing of attrition, as well as systematically documenting any adverse events, both related to the studied condition and to the intervention itself, such as VR-based interventions.

As a direct implication of our findings, the pooled dropout rate of 16% provides an important benchmark for planning future clinical trials. When calculating sample sizes, researchers should consider this rate to ensure adequate power despite anticipated losses. From a clinical standpoint, this moderate level of attrition suggests that VR interventions are generally feasible and acceptable in oncology settings, although paying attention to user engagement and tailored support is necessary. Identifying and addressing factors contributing to dropout may further improve adherence, especially in real-world deployments where monitoring is less intensive than in research contexts.

### 4.2. Future Research

Considering the gaps identified in this systematic review, we identified some needs that should be considered in future research on this topic. First, more studies are needed to evaluate how different VR modalities (immersive, semi-immersive, and non-immersive) affect not only pain outcomes but also participant dropout and adherence. These technological formats may differ significantly in terms of tolerability and engagement, which could influence retention in trials. Once a larger body of studies is available, future meta-analyses should compare dropout rates across VR types to identify which formats are more acceptable and sustainable for patients with cancer or for cancer survivors.

These comparisons should also consider the context of application, the primary objectives of the intervention (e.g., relaxation, physical rehabilitation, distraction), and patient characteristics such as age, disease stage, and symptom burden. Tailoring VR interventions to the specific needs and limitations of oncology populations may help to minimize dropout and enhance the real-world utility of these tools.

Second, future trials must improve adherence to reporting standards, particularly those outlined in the CONSORT guidelines [33]. It is essential to report the exact number of participants lost at each phase of the study, along with the specific reasons for dropout. This level of transparency will help identify common attrition patterns, whether due to physical deterioration, lack of interest, side effects, or logistical challenges, and ultimately support the development of more targeted and effective retention strategies. To facilitate future comparisons across VR modalities, trials should adopt minimum reporting standards that detail key features of the intervention, such as immersion level, content type, session duration and frequency, usability, and any adverse effects. Following established frameworks like the CONSORT guidelines for technology-based interventions can improve transparency and support higher-quality evidence synthesis.

Improved reporting is not only a matter of methodological rigor; it is also critical for preserving both internal and external validity in randomized controlled trials. High or unexplained dropout can undermine the reliability of findings and limit their applicability in clinical settings. By identifying and addressing the underlying causes of participant loss, future research can generate more robust, generalizable evidence and contribute to safer, more inclusive implementation of VR in oncology care.

### 4.3. Limitations

This review presents several limitations that must be considered when interpreting the findings. First, the number of randomized controlled trials included was limited, which reduced the statistical power of the meta-analysis and restricted the generalizability of the results. Consequently, the subgroup and meta-regression analyses may have been underpowered to detect relevant associations between study characteristics and dropout rates.

Second, a significant proportion of studies did not adequately report the reasons for participant attrition or the timing of these losses. This lack of detail hinders our ability to identify patterns with regard to dropout and to develop evidence-based strategies aimed at improving retention in future trials. Third, due to the scarcity of trials employing immersive VR, it was not possible to evaluate whether this modality leads to higher or lower dropout rates compared to non-immersive approaches.

In addition, substantial variability in the characteristics of the study populations introduces clinical heterogeneity that limits the extrapolation of findings to specific subgroups. Finally, the overall certainty of evidence, as assessed using the GRADE framework, was rated as very low, which underscores the need for caution in interpreting the results and highlights the importance of conducting additional high-quality trials in this field. Finally, the inclusion of both adult and pediatric populations, although methodologically justified by the scarcity of available trials, introduces additional clinical heterogeneity. Differences in pain perception, cognitive development, and interaction with VR technology may affect adherence differently across age groups. Future research should consider stratifying analyses by age or focusing on more homogeneous populations to enhance interpretability and applicability.

## 5. Conclusions

This systematic review and meta-analysis is the first to quantify dropout rates in randomized controlled trials using virtual reality (VR) interventions for pain management in individuals with cancer. The pooled dropout rate was 16%, with slightly lower rates observed in VR-based interventions compared to controls, although without statistically significant differences. These findings suggest that VR interventions generally achieve acceptable retention rates in oncology settings.

However, substantial heterogeneity across studies—including differences in VR modalities, comparator groups, patient populations, and reporting practices—limits the ability to draw definitive conclusions about the overall feasibility and acceptability of VR interventions. Moreover, limited reporting on reasons for dropout restricts our understanding of factors influencing participant attrition.

Despite these limitations, this review provides a valuable benchmark for future clinical trial design, particularly regarding sample size estimation and retention expectations. Future research should prioritize standardized reporting of dropout reasons and adherence metrics and investigate how specific VR characteristics and patient factors influence engagement. Strengthening methodological rigor and transparency will be essential to optimize the integration of VR technologies into supportive cancer care and improve patient outcomes. Addressing these gaps will contribute to safer and more efficient integration of VR technologies into supportive cancer care.

## Figures and Tables

**Table 1:** Methodological quality assessment.

Author (Year)	Item 1	Item 2	Item 3	Item 4	Item 5	Item 6	Item 7	Item 8	Item 9	Item 10	Item 11	Item 12	Item 13
Abdelmoniem et al., 2024 [12]	Y	Y	Y	Y	N	Y	Y	Y	Y	Y	Y	Y	Y
Basha et al., 2022 [13]	Y	Y	U	N	N	Y	Y	Y	Y	Y	Y	Y	Y
Erdős et al., 2023 [14]	Y	N	Y	N	N	Y	Y	Y	Y	Y	Y	Y	Y
Feyzioğlu et al., 2020 [15]	Y	Y	Y	N	N	Y	Y	Y	Y	Y	Y	Y	Y
Giannelli et al., 2024 [16]	Y	Y	U	N	N	Y	Y	Y	Y	Y	Y	Y	Y
Li et al., 2024 [17]	Y	Y	Y	N	N	Y	Y	Y	Y	Y	Y	Y	Y

Note: N: No; U: uncertain; Y: Yes. Items: 1. Was true randomization used when assigning participants to treatment groups? 2. Was allocation to treatment groups concealed? 3. Were treatment groups similar at the baseline? 4. Were participants blind to treatment assignment? 5. Were those delivering the treatment blind to treatment assignment? 6. Were treatment groups treated identically with the exception of the intervention of interest? 7. Were outcome assessors blind to treatment assignment? 8. Were outcomes measured in the same way for treatment groups? 9. Were outcomes measured in a reliable way? 10. Was follow up complete and if not, were differences between groups in terms of their follow-up adequately described and analyzed? 11. Were participants analyzed in the groups to which they were randomized? 12. Was appropriate statistical analysis used? 13. Was the trial design appropriate and any deviations from the standard RCT design (individual randomization, parallel groups) accounted for in the conduct and analysis of the trial?

**Table 2:** Data extraction.

Study	Population		Intervention	Comparation	Outcomes			
	N CG EG	Type of Cancer Gender Mean Age (Years)	Type (Duration)	Type (Duration)	% Overall Retention	% Dropout	Reason for Dropouts	Adverse Events
Abdelmoniem et al., 2024 [12]	N: 54 CG: 27 EG: 27	Post-operative breast cancer F 100% M 0% CG: 48 ± 4.60 GE: 47 ± 3.94	Exercises using VR technology and standard treatment (3/week; 8 weeks). Exercises using VR technology (Pablo© Handle Training): 5 VR games (3 min/game; 15 min). Non-immersive virtual reality. Standard treatment: upper limb exercises (2 sets, 15 repetitions 15 min) and electronic intermittent compression therapy.	Standard treatment: upper limb exercises (2 sets, 15 repetitions 15 min) and electronic intermittent compression therapy (3/week; 8 weeks).	N: 74.07% (40/54) CG: 77.78% (21/27) CG: 70.37% (19/27)	N: 25.93% (14/54) EG: 22.22% (6/27) CG: 29.63% (8/27)	Not indicated	None
Basha et al., 2022 [13]	N: 60 CG: 30 EG: 30	Unilateral breast cancer-related lymphedema F 100% M 0% CG: 52.07 ± 7.48 GE: 48.83 ± 7.0	VR Kinect-based games and complex decongestive physiotherapy in small groups (1–4 women) supervised by a physiotherapist (1 session/day, 5 days/week, 8 weeks) VR Kinect-based games (Xbox Kinect): active movements of all joints of the upper limbs. Non-immersive virtual reality Complex decongestive physiotherapy: manual lymphatic drainage, compression bandages, skin care, and exercises.	Upper limb exercises and complex decongestive physiotherapy in small groups (1–4 women) supervised by a physiotherapist (1 session/day, 5 days/week, 8 weeks) Stretching and strength exercises in upper limb (2–3 sets; 10–12; 2 min rest allowed between sets) Complex decongestive physiotherapy: manual lymphatic drainage, compression bandages, skin care, and exercises.	N: 96.67% (58/60) CG: 93.33% (28/30) EG: 100% (30/30)	N: 3.33% (2/60) CG: 6.67% (2/30) EG: 0% (0/30)	Not indicated	Not indicated
Erdős et al., 2023 [14]	N: 35 CG: 14 EG: 21	Pediatric cancer patients underwent chemotherapy F 27.59% M 72.41% N: 15 ± 2.44	VR game (during the chemotherapy process). VR game (A Night Sky): Immersive virtual reality.	Playing a mobile game according to participants’ own preferences (during chemotherapy process)	N: 82.86% (29/35) CG: 71.43% (10/14) EG: 90.48% (19/21)	N: 17.14% (6/35) CG: 28.57% (4/14) EG: 9.52% (2/21)	CG: declined to participate (n = 4) EG: declined to participate (n = 2)	Not indicated
Feyzioğlu et al., 2020 [15]	N: 40 CG: 20 EG: 20	Breast cancer patients who underwent unilateral mastectomy with axillary lymph node dissection and were receiving adjuvant therapy F 100% M 0% CG: 51 ± 7.06 GE: 50.84 ± 8.53	VR-exercise using Xbox Kinect-based games (45 min/session; 2 sessions/week, 6 weeks) VR-exercise: functional and strength exercises for upper limbs using a VR system (Xbox Kinect-based games). Supervised by an experienced physiotherapist. Non-immersive virtual reality.	Standard physiotherapy (45 min/session; 2 sessions/week, 6 weeks) Standard physiotherapy: exercises not using any VR system. Supervised by an experienced physiotherapist.	N: 90% (36/40) CG: 85% (17/20) EG: 95% (19/20)	N: 10% (4/40) CG: 15% (3/20) EG: 1% (1/20)	EG: declined to participate (n = 1) CG: new metastasis focus (n =1), declined to participate (n = 1), chemotherapy side effect (n = 1)	None found
Giannelli et al., 2024 [16]	N: 56 CG: 26 EG: 27	Advanced cancer patients F 58% M 42% N: 55.7 ± (10.7) CG: 58.23 ± 8 GE: 53.2 ± 12.4	VR headset with interactive and non-interactive content (4 days; to avoid forced use of the device, the investigator did not specify a minimum or maximum usage time or number of sessions; supervised by psychologists) VR headset: interactive content based on a basic three-level skill game called ‘Yuma’s World’. Non-interactive content based on 10 immersive 360° videos with natural and relaxing scenarios. Immersive virtual reality.	Tablet (TAB) which played 10 non-interactive 2D videos depicting natural and relaxing scenarios. (4 days; to avoid a forced use of the device, the investigator did not specify a minimum or maximum usage time or number of sessions)	N: 80.36% (45/56) CG: 84.62% (22/26) EG: 74.07% (20/27)	N: 19.64% (11/56) CG: 15.38% (4/26) EG: 25.92% (7/27)	All dropouts were because of no autonomous device use	None found
Li et al., 2024 [17]	N: 327 CG: 125 EG: 163	Breast cancer undergoing chemotherapy F 100% M 0% MA: 54.3 ± 9.6 CG: 53.6 ± 9.4 GE: 55.2 ± 9.7	Relax VR intervention (during chemotherapy intervals/breaks and 15–20 min 1 to 2 sessions/week; 12 weeks) Relax VR intervention: two parts. Firstly, using a headset (VIVES110) and secondly using a hand controller. In the hospital supervised by nurses. Immersive virtual reality.	Traditional care during chemotherapy. They refrained from the beginning any VR treatment.	N: 75.23% (246/327) CG: 76.23% (125/164) EG: 74.23% (121/163)	N: 24.77% (81/327) CG: 23.78% (39/164) EG: 25.77% (42/163)	EG: intervention times < 12 (n = 10), physical problems (n = 15), loss of interest (n = 8), other reasons (n = 9) CG: Physical problems (n =17), loss of interest (n = 12), other reasons (n = 10)	Not indicated

Notes: CG: control group, EG: experimental group, F: females, M: males; N: overall sample; VR: virtual reality.

**Table 3:** Univariate meta-regression analysis of covariates of the meta-analysis of outcomes measured in participants with cancer and pain.

Covariate (k)	Coefficient β (95% CI) ^1^	*p*-Value
Age (5)	0.001 (−0.06 to 0.09)	0.64
Cancer type:		
Breast (4)	−0.26 (−1.38 to 0.85)	0.55
Experimental interventions:		
Non-immersive virtual reality (3)	−0.77 (−2.30 to 0.77)	0.23
Immersive virtual reality (3)	0.08 (−0.54 to 0.71)	0.73
Frequency of interventions (4)	0.27 (−2.71 to 3.25)	0.74
Number of female participants (6)	0.001 (−0.004 to 0.01)	0.41
Number of male participants (6)	0.004 (−0.08 to 0.09)	0.91
Number of sessions (4)	0.11 (−0.06 to 0.27)	0.11
Number of weeks of interventions (4)	0.19 (−0.13 to 0.50)	0.12
Sample size (6)	0.002(−0.004 to 0.009)	0.39

^1^ Mixed model effect-based meta-regression; Only moderators included in at least three studies were assessed. Redundant moderators were avoided. k: number of studies analyzed. Statistical significance (bold) *p* < 0.05.

**Table 4:** Certainty of evidence (GRADE) for the odds ratio meta-analyses that compare dropout rates between experimental and control intervention.

Summary of Findings			Certainty of Evidence Based on the GRADE Approach					
Outcome	Studies (n/k)	Participants (N)	Risk of Bias	Inconsistency	Indirectness	Imprecision	Level of Evidence	Importance
Odds ratio meta-analysis	6	569	Very Serious ^1^ (−2)	No	Very Serious ^2^ (−2)	No	Very Low	Critical

Note: GRADE = Grading of Recommendations Assessment, Development and Evaluation. ^1^ Downgraded by two levels due to most of the information coming from RCTs with a high risk of bias with potential limitations that are likely to lower confidence in the estimations of effects. ^2^ Downgraded by two levels due to the significant heterogeneity between experimental interventions and measurement of outcomes.

**Figure A1:**
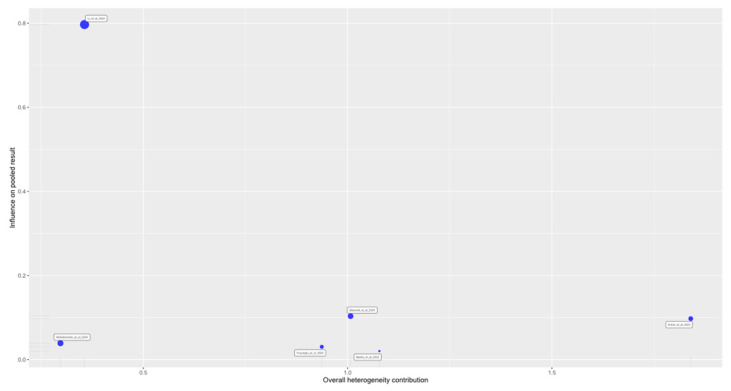
Baujat plots. Abdelmoniem et al., 2024 [12], Basha et al., 2022 [13], Erdős et al., 2023 [14], Feyzioğlu et al., 2020 [15], Giannelli et al., 2024 [16], Li et al., 2024 [17].

**Figure A2:**
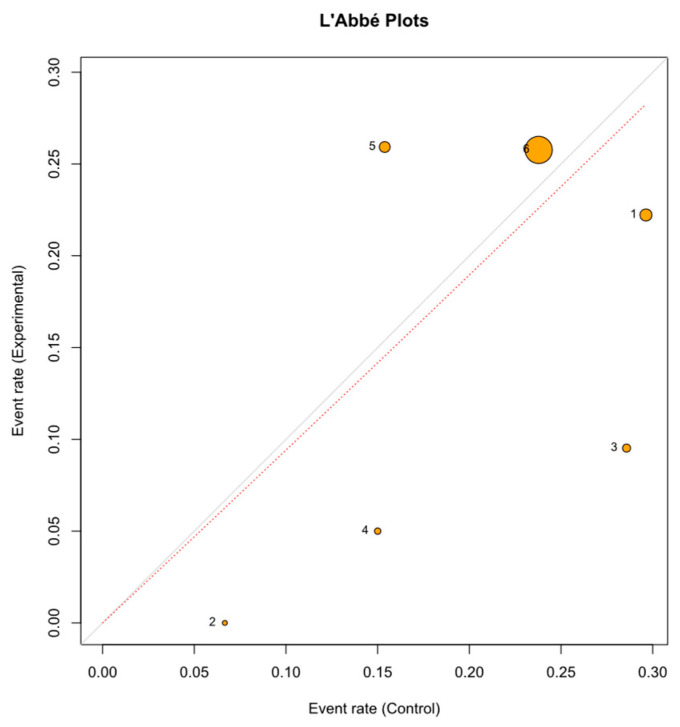
L’Abbé plots. L’abbe plot shows the trend of the included study (dot) to present a higher (upper the line) or lower (below the line) drop of participants.

**Figure A3:**
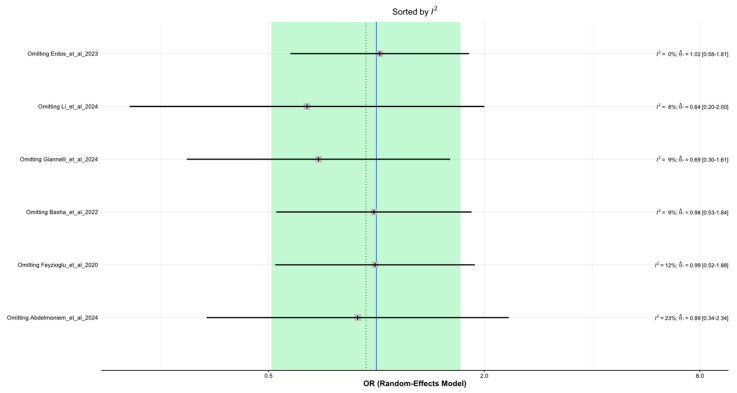
Leave-one-out diagnostics. Abdelmoniem et al., 2024 [12], Basha et al., 2022 [13], Erdős et al., 2023 [14], Feyzioğlu et al., 2020 [15], Giannelli et al., 2024 [16], Li et al., 2024 [17].

**Figure A4:**
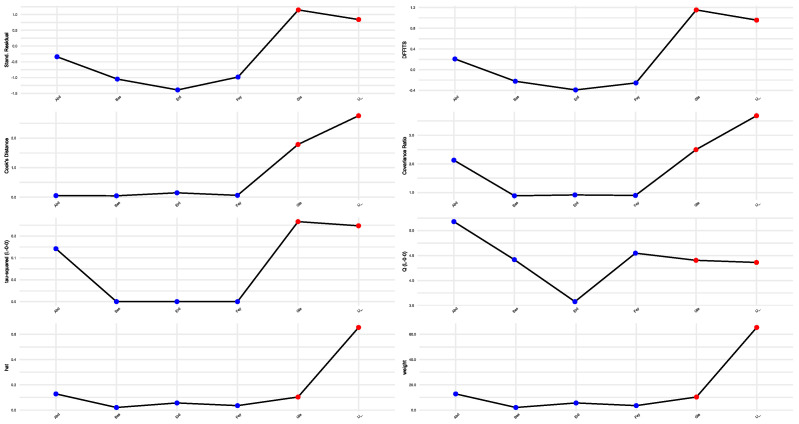
Influence plots. Influence plot shows in red color the studies that influence more in the pooled size effect.

## Data Availability

No new data were created or analyzed in this study. Data sharing is not applicable to this article.

## Supplementary Materials

The following supporting information can be downloaded at https://www.mdpi.com/article/10.3390/healthcare13141708/s1, Supplementary File S1. Extended search strategy used in PubMed. Supplementary File S2. Detailed description of the selection process. Supplementary File S3. Forest plots from the different subgroup analysis. Supplementary File S4. Detailed description of the selection process. Refs. [34,35,36,37,38,39,40] are cited in the Supplementary Materials file.

## Abbreviations

The following abbreviations are used in this manuscript:

VR	Virtual Reality

## Appendix A

### Appendix A.1

Figure A1, Figure A2, Figure A3 and Figure A4 show all sensitivity analyses: Baujat plots, L’Abbé plots, leave-one-out diagnostics, and influence plots.
