# Supplementary material for: Dropout Rate of Participants with Cancer in Randomized Clinical Trials That Use Virtual Reality to Manage Pain—A Systematic Review with Meta-Analysis and Meta-Regression
Source: Healthcare (Basel). 2025 Jul 16;13(14):1708. doi: 10.3390/healthcare13141708 (PMC12294435; doi:10.3390/healthcare13141708)
Supplement: Supplementary file 1 [file healthcare-13-01708-s001.zip › healthcare-3600742-supplementary.pdf]

Supplementary File S1. Extended search strategy used in PubMed

Search terms: #1 cancer, #2 neoplasms #3 virtual reality #4 virtual reality exposure therapy #5 pain #6 pain management.

Search: **("cancer"[Title/Abstract] OR "neoplasms"[MeSH Terms]) AND ("virtual reality"[Title/Abstract] OR "virtual reality exposure therapy"[MeSH Terms]) AND ("pain"[Title/Abstract] OR "pain management"[MeSH Terms])**

Filters: **Clinical Trial, Randomized Controlled Trial**

(("cancer"[Title/Abstract] OR "neoplasms"[MeSH Terms]) AND ("virtual reality"[Title/Abstract] OR "virtual reality exposure therapy"[MeSH Terms]) AND ("pain"[Title/Abstract] OR "pain management"[MeSH Terms])) AND (clinicaltrial[Filter] OR randomizedcontrolledtrial[Filter])

Supplementary File S2. Detailed description of the selection process.

The selection process was performed following the PRISMA recommendations. A total of 239 records were identified, all of them in databases: PubMed (n = 32), Web of Science (n = 33), Scopus (n = 131) and CINAHL (n=3). After removed duplicated records (n = 50), studies were screened (n = 180). A percentage of them (n = 167) were excluded by Title / Abstract. We accessed to review full texts for elegibility in the next step of selection process (n = 13). Seven of them were excluded. Table S1 shows the references and the reasons of exclusion of each one. Finally, 6 studies [11-16] were included in the qualitative and in the quantitative synthesis. Figure 1 show in the manuscript shows the flow diagram.

Table S1. Results excluded by full-text.

STUDY (n = 7)	EXCLUDED BY	REASON
Chuan et al., 2023	Comparison	The comparison is based on application of VR intervention
Cîmpean et al., 2017	Type of study	The groups were not randomized.
Ioannou et al., 2022	Intervention	Measurements are made over a very short period of time, so studying dropout is pointless.
Saeidi et al., 2023		
Tennant et al., 2020		
Wong et al., 2021		
Mogahed et al., 2024	-	Data was not available.

Chuan, A.; Hatty, M.; Shelley, M.; Lan, A.; Chow, H.; Dai, E.; Haider, S.; Bogdanovych, A.; Chua, W. Feasibility of virtual reality-delivered pain psychology therapy for cancer-related neuropathic pain: a pilot randomised controlled trial. *Anaesthesia* **2023**, *78(4)*, 449–457. https://doi.org/10.1111/anae.15971

Cîmpean, A. I. A pilot study to compare cognitive behavioral therapy with virtual reality vs. Standard cognitive behavioral therapy for patients who suffer from cervical cancer. *Journal of Evidence-Based Psychotherapies* **2019**, *19*(1), 115–127. https://doi.org/10.24193/jebp.2019.1.7

Ioannou, A.; Paikousis, L.; Papastavrou, E.; Avraamides, M. N.; Astras, G.; Charalambous, A. Effectiveness of Virtual Reality Vs Guided Imagery on mood changes in cancer patients receiving chemotherapy treatment: A crossover trial. *European Journal of Oncology Nursing* **2022,** *61*, 102188. https://doi.org/10.1016/j.ejon.2022.102188

Saeidi, M.; Bostanabad, M. A.; Jabraeili, M. The Effect of Virtual Reality and Hugo Point Massage on the Pain and Anxiety of School-aged Children with Cancer: Crossover Clinical Trial. *Open Nursing Journal* **2023**, *17*. https://doi.org/10.2174/0118744346248844231003110642

Tennant, M.; Youssef, G. J.; McGillivray, J.; Clark, T.-J.; McMillan, L.; McCarthy, M. C. Exploring the use of Immersive Virtual Reality to enhance Psychological Well-Being in Pediatric Oncology: A pilot randomized controlled trial. *European Journal of Oncology Nursing* **2020**, *48*, 101804. https://doi.org/10.1016/j.ejon.2020.101804

Wong, C. L.; Li, C. K.; Chan, C. W. H.; Choi, K. C.; Chen, J.; Yeung, M. T.; Chan, O. N. Virtual Reality Intervention Targeting Pain and Anxiety Among Pediatric Cancer Patients Undergoing Peripheral Intravenous Cannulation. *Cancer Nursing* **2021**, *44(6)*, 435–442. https://doi.org/10.1097/NCC.0000000000000844

Mogahed, H. G.; Hamoda, R. E.; Elkalla, R. A. Virtual reality on pain and anxiety after modified radical mastectomy in menopause. *Research Journal of Pharmacy and Technology* **2024**, *17(4)*, 1657–1661. https://doi.org/10.52711/0974-360X.2024.00262

Supplementary File S3. Forest plots from the different subgroup analysis

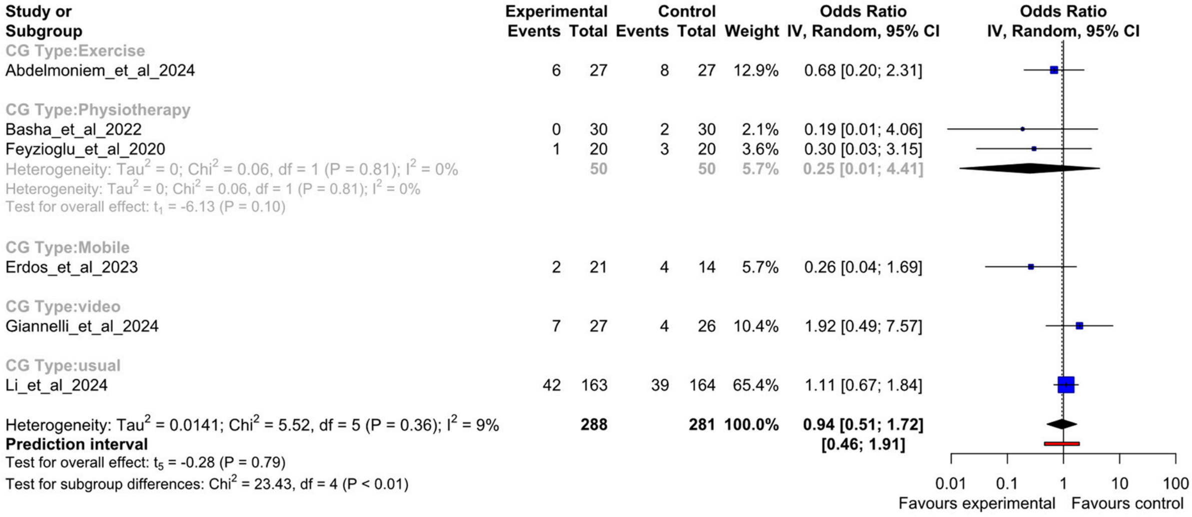

Figure S1. Forest plots in control subgroup

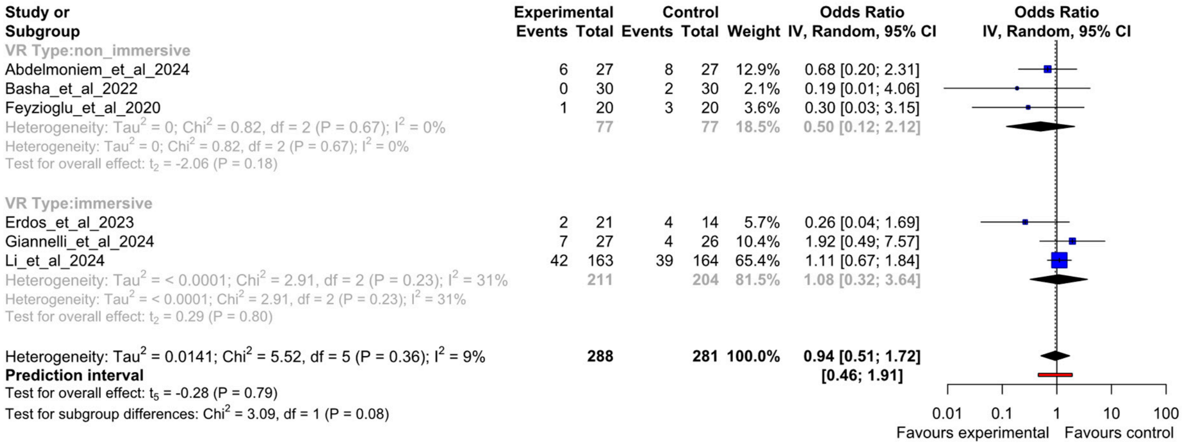

Figure S2. Forest plots in experimental subgroup

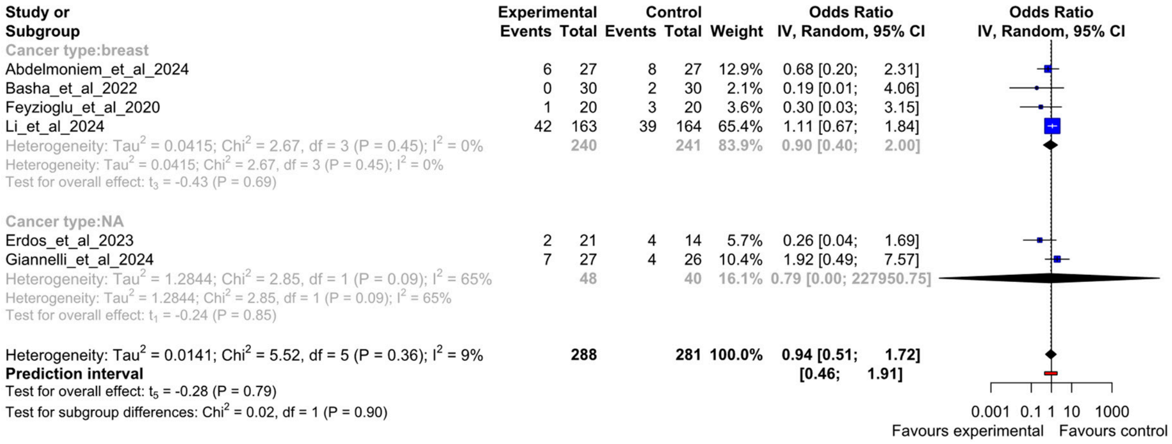

Figure S3. Forest plots in cancer type subgroup

Supplementary File S4. Detailed description of the selection process.

Table S2. Results excluded by full-text.

Study	Dropout Reasons Reported	Summary of Reported Reasons
**Abdelmoniem et al., 2024**	No	Not specified
**Basha et al., 2022**	No	Not specified
**Erdos et al., 2023**	Yes	Declined to participate (EG: n=2, CG: n=4)
**Feyzioğlu et al., 2020**	Yes	EG: declined (n=1); CG: new metastasis, declined, chemo side effects (n=3)
**Giannelli et al., 2024**	Yes	All due to lack of autonomous use of device (EG: n=7, CG: n=4)
**Li et al., 2024**	Yes	EG: Intervention times < 12 (n=10), physical problems (n=15), loss of interest (n=8), other reasons (n=9)<br>CG: Physical problems (n =17), loss of interest (n=12), other reasons (n=10)
